# Outreach training and supportive supervision for malaria case management in Zambia: the effects of focused capacity building on indicators of diagnostic and clinical performance

**DOI:** 10.1186/s12936-018-2589-6

**Published:** 2018-11-28

**Authors:** Matt Worges, Nicole Whitehurst, Emanuel Yamo, Hawela Moonga, Joshua Yukich, Luis Benavente

**Affiliations:** 1 Medical Care Development International, Silver Spring, MD USA; 2 US President’s Malaria Initiative Improving Malaria Diagnostics Project, Washington, DC USA; 3 Tulane University School of Public Health and Tropical Medicine, New Orleans, LA USA; 4 National Malaria Elimination Centre, Ministry of Health, Lusaka, Zambia; 5 Department of Tropical Medicine, Center for Applied Malaria Research and Evaluation, Tulane University School of Public Health and Tropical Medicine, New Orleans, LA USA

**Keywords:** Malaria, Diagnostics, Clinical, Case management, Supportive supervision, Capacity building

## Abstract

**Background:**

Accurate diagnosis of malaria and reduced reliance on presumptive treatment are crucial components of quality case management. From 2008 to 2012, the Improving Malaria Diagnostics project, in collaboration with the Zambia National Malaria Control Centre, implemented an external quality assurance scheme partially comprised of outreach training and supportive supervision (OTSS) in an effort to improve malaria case management across a spectrum of health facilities performing laboratory-based malaria diagnostics. OTSS assessments were conducted by project-trained laboratory and clinical supervisors on a regular basis and measured changes in health facility staff performance over time. Standardized supervision tools were used for data collection and guided OTSS teams to assess health facility infrastructure, record keeping practices, stores of supplies and consumables, good laboratory practices, and staff adherence to guidelines for the case management and diagnosis of suspected malaria cases via direct observations or record reviews. The structure of OTSS also allowed supervisors to provide ongoing support to clinicians and laboratory staff through regular mentoring and on-the-job training.

**Results:**

This analysis included 88 laboratories and 64 clinics each with four repeated supervisory assessments. Over the course of the project there were significant declines in the number of laboratories experiencing stock-outs of microscopy reagents/consumables (p < 0.001) and significant increases in the number of laboratories instituting the use of microscopy positive controls (p < 0.01), conducting parasite counting (p < 0.05), and converting from a semi-quantitative to a quantitative parasite counting methodology (p < 0.001). Performance in malaria diagnostic and clinical practices [i.e. RDT use (mean(diff) = 14.3%, p < 0.001), blood slide preparation (mean(diff) = 14.7%, p < 0.001), blood slide staining and reading (mean(diff) = 14.0%, p < 0.001), fever case management (mean(diff) = 7.3%, p < 0.01)] and prescriber adherence to negative diagnostic test results (mean(diff) = 7.2%, p < 0.05) showed modest, but significant gains from assessment 1 to assessment 4.

**Conclusion:**

The external quality assurance scheme provided periodic representations of clinical and laboratory staff performance. OTSS-enrolled health facilities demonstrated improvements to malaria diagnostic skills, adoption of laboratory best practices, strengthened fever case management practices, and improved prescriber adherence to negative malaria test results.

**Electronic supplementary material:**

The online version of this article (10.1186/s12936-018-2589-6) contains supplementary material, which is available to authorized users.

## Background

In 2008, the World Health Organization (WHO) reported that the proportion of people treated for malaria with a confirmed diagnosis was low in Africa compared to other regions of the world [1]. In response to this and due to the high cost of artemisinin-based combination therapy (ACT) at the time, the WHO released recommendations urging that clinical suspicion of malaria be confirmed with a parasitological diagnosis before treatment with anti-malarials wherever possible [2]. In line with these recommendations, the Republic of Zambia called for parasite-based diagnosis of malaria in order to curb its clinical misdiagnosis and associated over prescription of anti-malarial drugs [3]. Alongside the adoption of such recommendations, improving the quality of malaria case management and diagnostic services is essential if health outcomes are to be significantly improved [4].

As accurate diagnosis of malaria and reduced reliance on presumptive treatment are crucial components of quality case management, the Zambia National Malaria Control Centre (NMCC), now known at the National Malaria Elimination Centre, with support from the international community, began implementation of an outreach training and supportive supervision (OTSS) programme in 2009 that was designed to improve malaria case management and diagnostics. Such programmes promote quality within and across health facilities by strengthening communication and relationships, focusing on the identification and resolution of problems, helping to optimize the allocation of resources, and empowering health providers to monitor and improve their own performance [5].

Appropriate diagnosis of malaria requires an organized health facility infrastructure with functional equipment, regular supply of consumables and reagents, and trained technicians who operate within a quality control framework [6]. Improving malaria diagnosis, therefore, requires a multi-faceted approach [7] and supervision/training programmes must focus not only on building technician capacity to conduct malaria microscopy and rapid diagnostic tests (RDTs), but also take care to address issues concerning stock-outs of essential supplies and consumables, equipment maintenance, institutionalization of internal quality assurance (IQA) practices, and, to the extent possible, management of diagnostic practices within environments prone to insecure infrastructure. In addition to microscopy, malaria RDTs are widely available for use in Zambia, first being introduced on a pilot basis in 2005, with sufficient supplies regularly in stock across a majority of health facilities [8, 9]. A cross-sectional cluster sample survey of 104 health facilities across 4 districts of Zambia found that 63% had RDTs available and 73% could perform either microscopy or RDTs [6]. Further, the NMCC has encouraged the use of RDTs by non-laboratory staff and in facilities where microscopy is not available [10]. However, malaria diagnostics have not always been routinely used and adherence to test results has been previously reported at low levels in public health settings resulting in erroneous prescription of anti-malarials [6, 9, 11] particularly leading up to the time when the OTSS programme was first being implemented.

Prescriber adherence practices for malaria (i.e. prescription of ACT based on parasitological diagnosis via microscopy or RDTs) are likely to be influenced by a number of factors including reliance on clinical acumen as well as clinician confidence in malaria diagnostic test results [12]. The latter may be compromised due to either real or perceived poor performance of laboratory staff members responsible for conducting these tests which, in turn, contributes to the presumptive treatment of febrile patients by prescribers [13]. Indeed, a 2007 cross-sectional study conducted in six districts of Tanzania revealed that only 41.0% (25/61) of interviewed clinicians considered malaria microscopy results from their respective laboratories to be reliable [14]. Further, the quality of treatment provided at the point of care has been reported to be sub-optimal during the period preceding the implementation of the OTSS programme in Zambia [9, 15].

Focused capacity building via supportive supervision or intensive training can serve to improve ACT prescription practices and may even work to encourage clinic reliance on laboratory test results. Indeed, a study of monthly malaria case management supportive supervision for 55 primary health care workers in Jos, Nigeria found a non-significant increase in the proportion of health care workers waiting for laboratory test results before making a diagnosis of malaria (29.1% at pre-intervention and 58.8% at post-intervention within a 3-month interval) [16]. Additionally, a randomized controlled trial conducted in Tanzania concluded that training in microscopy-based diagnosis of malaria at study-enrolled health facilities reduced prescription of anti-malarial drugs and appeared to have an overall positive impact on management of non-malaria fevers [17].

A body of evidence exists to suggest that supportive supervision and audit with feedback are generally quite effective at improving health worker performance and productivity [7, 18–22]. Additionally, multifaceted interventions that are designed to address multiple determinants of performance are more likely to improve outcomes than single interventions alone such as the dissemination of written guidelines [7, 23, 24]. However, many of the strategies employed by researchers to evaluate the effects of supportive supervision yield mixed results [7]. Further, a number of studies assessing malaria case management performance via interventional studies have focused exclusively on either laboratory [25–28] or clinical personnel [16]. Few studies [29, 30] have focused on assessing both clinical and laboratory malaria case management practices and diagnostic performance in a concurrent manner—the aim of the OTSS programme. The results of the OTSS programme add to the evidence base of supportive supervision initiatives by broadening the scope of outcome measures related to the practice. The primary outcomes assess adherence to guidelines and protocols for malaria parasitological diagnosis and fever case management with secondary outcomes focused on adoption of clinical and laboratory best practices. The results also provide a general overview of the state of malaria case management practices across a selection of health facilities in Zambia during OTSS implementation.

### Project background

The Improving Malaria Diagnostics (IMaD) project was a 5-year partnership (2008–2012) funded by the US Agency for International Development under the US President’s Malaria Initiative. In partnership with host country national malaria control programmes, IMaD was tasked with developing a programme to identify weaknesses and gaps in malaria case management and diagnostic practices and respond to them through technical training, regular mentoring and monitoring of health facility staff, enhancement of quality assurance systems, policy development and implementation strategies, and guidance on procurement of laboratory consumables and essential equipment. Accordingly, the IMaD project developed an external quality assurance scheme, of which OTSS was a component, designed to support malaria case management and diagnostics across a spectrum of health facilities with demonstrated ability to conduct malaria microscopy in a laboratory setting. In Zambia, the OTSS programme was established in partnership with the NMCC and programme implementation was carried out by cadres of project-trained laboratory and clinical supervisors whose primary objectives were to provide periodic representations of health facility operations and performance of malaria-specific clinical and laboratory practices. The structure of OTSS also allowed supervisors to provide ongoing support to clinicians and laboratory staff through regular mentoring and on-the-job training.

### Programme implementation

In August 2009, clinical and laboratory supervisors were invited to a 1-week training course covering the OTSS conceptual model, concepts of quality assurance, and use of the OTSS supervisory checklists. Prior to OTSS assessments, laboratory supervisors were invited to a malaria microscopy training course where their skills in parasite detection, species identification, and parasite quantification were refreshed and assessed for competency [31]. Additionally, clinical supervisors were invited to attend a 3-day clinical case management refresher training.

Standardized checklists were developed by the IMaD technical team, OTSS supervisors, and Ministry of Health (MoH) and malaria control programme central-level staff from IMaD-supported countries. Checklists were used to measure changes in health facility staff performance over time and to collect diagnostic and clinical data on patient management. In Zambia, project-developed checklists were first revised by the MoH-affiliated case management working group before a core group of clinical and laboratory supervisors were trained on their content. The checklists were then field tested in urban, peri-urban, and rural settings across various facility types (i.e. central and provincial hospitals and health centers with functioning laboratories). Additional revisions were made to the checklists after field testing followed by MoH sign-off on their use for the OTSS programme. In accordance with their expertise, supervisors used laboratory- or clinic-specific checklists during the OTSS assessments. In an effort to encourage communication across both cadres of OTSS supervisors, the project held annual lessons learned meetings where teams discussed progress to date as a group and had the opportunity to debrief and share best practices in an open and structured fashion.

Facilities were enrolled into the OTSS programme in a purposive manner resulting in a non-probability sample. Only facilities known to have staff performing malaria microscopy within a functional laboratory were considered for inclusion in the OTSS program. An additional selection criterion focused on including those facilities with higher patient throughput as determined by local experts (i.e. NMCP staff and senior OTSS supervisors). It is important to note, however, that the Government of Zambia encouraged bringing the OTSS programme to scale across all provinces. Eligible facilities were enrolled in a rolling fashion throughout the project’s timeframe. In addition to health centre enrollment, the OTSS programme conducted assessments at district, provincial, and central hospitals. Health posts were excluded from the OTSS programme due to existing supervision structures targeting community health workers. OTSS supervisors visited the same health facilities during each round of assessments in order to build rapport with the staff they were supporting.

## Methods

### Supervision programme

OTSS supervisors collected basic health facility information related to infrastructure, general functionality, and availability of resources. Additionally, supervisors assessed the occurrence of stock-outs of essential malaria microscopy consumables and reagents at some point in the 3 months preceding each supervisory visit. They also investigated patient records to assess prescriber adherence to negative malaria tests (supervisors were requested to investigate up to ten patient records from which a score could be derived by taking a simple proportion of test-negative patients with no evidence of anti-malarial prescriptions against all investigated records). Supervisors investigated clinical registers/log books to ensure they were properly completed and maintained; however, patient registers themselves were not wholly standardized across facilities. Some facilities used notebooks or blank registers in which they created columns for specific elements they wished to record while others had MoH-issued registers with pre-printed column headings. Regardless of register type, OTSS supervisors were instructed to conduct the following: (1) confirm if daily outpatient/inpatient register book is available, (2) confirm that information is correctly recorded, (3) confirm that information is completely recorded, (4) confirm that the register is up to date, and (5) confirm that there is a filing/archiving system in place for exhausted registers. Further, at each visit, OTSS supervisors observed clinical staff as they conducted febrile patient consultations as well as laboratory technicians as they conducted malaria microscopy and RDT procedures.

Additional file 1 provides the detailed steps/tasks against which facility staff were observed and scored.

Following data collection and observation activities, OTSS supervisors provided constructive feedback to health facility staff on methods to improve performance for malaria case management and diagnostics procedures which also encompassed advice on basic health facility functioning, including patient flow through the health facility; maintaining appropriate stores of reagents, consumables, and pharmaceuticals; institutionalization of IQA and good laboratory practices; appropriate clinical documentation; and reporting of malaria case data. Aside from corrective actions and mentorship at the time of the last OTSS visit, no experimental manipulation directly preceded data collection and as such data generated from the OTSS programme should be considered observational in nature and based on programmatic activities. Additionally, due to funding and time constraints, the OTSS programme was not able to assess health facility staff performance beyond planned supervisory visits.

### Data collection and management

Supervisory assessments were conducted on a regular basis with the use of standardized checklists. All health facility visits for the first round of assessments occurred within the same period of time, but the second assessment for clinical OTSS was delayed due to scheduling difficulties; otherwise, general timing of laboratory and clinical assessments was synchronized. Once the OTSS assessments were concluded the completed checklists were sent by courier to Lusaka where data were single entered by locally trained staff using a Microsoft Access database (Microsoft Corporation, Redmond, Washington, US). Data validation measures and subsequent analyses were conducted by project staff. Paper-based data collection was used for OTSS assessments.

### Statistical analysis

Data were cleaned and analysed using Stata 14 (StataCorp, 2015. Stata Statistical Software: Release 14. College Station, TX: StataCorp LP) and Excel 2013 (Microsoft Corporation, Redmond, Washington, US). The final clinical and laboratory data sets were obtained by restricting IMaD Zambia OTSS assessment records to only those facilities having four consecutive assessments where the first assessment represented the first contact between OTSS teams and the facility. During each OTSS assessment, supervisors were instructed to conduct up to three repeated observations for each laboratory or clinical practice in the checklist. Only the first observation scores for each observed practice from the first and fourth OTSS assessment visits were used for analysis, however. OTSS supervisors may have provided corrective action after the first observation potentially leading to improved performance for the second and third observations. This positioned the initial observation as a better indication of actual performance at the time of the OTSS assessment. Analysis of prescriber adherence to negative malaria test results was conducted for health facilities with at least five out of ten investigated and properly documented patient records. Unweighted proportions were calculated for a number of descriptive variables. Bivariate analyses (paired *t*-tests) were conducted to characterize performance gains in mean scores from one assessment to the next and from OTSS assessment 1 to assessment 4. McNemar’s test of independence was conducted on binary paired data for selected laboratory and clinical characteristics in order to assess marginal homogeneity. Three separate, adjusted regression models evaluating fever case management practices, malaria microscopy blood slide staining/reading performance, and RDT use performance were fitted using random-effects generalized least squares (GLS) linear regression. The imputation method of last observation carried forward was performed for the variables contained within these three regression models.

Bivariate regression analyses were first run to obtain unadjusted point estimates and variables with p-values < 0.1 were included in the adjusted models provided pairwise correlation tests did not detect appreciable multicollinearity among covariates (i.e. correlation coefficients ≥ 0.55). The ‘xtset’ command in Stata was used to designate panel (health facility unique identifier) and time (OTSS assessment number) variables and the ‘xtreg’ command was used to run the regression analyses.

## Results

The health facilities used in this analysis represented all 10 provinces of Zambia. A total of 91 unique facilities were included across both the laboratory and clinical data sets, just over two-thirds (61/91; 67.0%) of which underwent joint clinical and laboratory OTSS assessments (i.e. OTSS supervisors assessed clinical and laboratory staff and procedures from the same health facility). OTSS assessments generally occurred every 3–6 months for the facilities included in the current analysis and a facility’s total time undergoing these assessments rarely exceeded 2 years spanning a mean of 485 days for clinical OTSS (n = 61 health facilities) and 483 days for laboratory OTSS (n = 79 health facilities).

The laboratory data set included a total of 88 health facilities (36 health centers and 52 provincial/district hospitals), 68.2% (60/88) of which were in urban settings. Laboratory facilities were primarily government owned (66/88; 75.0%) as opposed to managed by faith-based organizations (22/88; 25.0%). An average of 3.5 functional microscopes with which malaria microscopy could be performed were noted for each assessed laboratory facility. At the time of the first OTSS assessment, when the majority of facilities were assessed for availability of reference materials, 71.4% (50/70) of all laboratories were missing key malaria diagnostic standard operating procedures and over half (37/67; 55.2%) did not have all recommended malaria diagnostic bench aids available for use by laboratory staff. Facility-level diagnostic turnaround time, the amount of time taken for laboratory staff to work through a diagnostic procedure, was assessed during the first OTSS assessment and averaged 45.6 min for malaria microscopy (n = 66) and 23.5 min for RDTs (n = 67). Turnaround time as measured here does not represent the amount of time elapsed between clinician request and receipt of test results.

The clinical data set was comprised of 64 facilities (41 provincial/district hospitals and 23 health centers), the majority of which were public (50/64; 78.1%) and urban (48/64; 75.0%). Nearly all (61/64; 95.3%) health facilities that underwent clinical OTSS also underwent laboratory OTSS. At the time of the first OTSS assessment and among only those facilities without missing data points, all health centres (21/21; 100.0%) and 73.2% (30/41) of hospitals had malaria case management and treatment guidelines available for use by clinicians. During the first OTSS assessment, all reporting facilities were found to have had at least 1 stethoscope (63/63; 100%) and at least 1 thermometer (60/60; 100%). An assessment of anti-malarial and drug stores at the time of the first OTSS visit showed that at least 95% (61/64) of all clinical facilities described their stocks of commonly used analgesics/antipyretics and malaria treatment drugs as always or usually available. Facility stock-outs of Zambia’s first-line anti-malarial, artemether–lumefantrine, were rare for both hospitals and health centres with only one health facility and two hospital clinics reporting stock-outs across all four assessments.

There were 60 clinics (38 hospitals and 22 health centres) with at least five patient records investigated for prescriber adherence to negative malaria tests (either microscopy or RDT) at both the first and last OTSS assessments. A mean of 9.9 and 9.8 patient records were investigated per clinic at these assessment points, respectively.

Table 1 shows the summary categories against which laboratory and clinical staff were assessed during OTSS observations of malaria microscopy, RDT use, and fever case management. The mean number of correctly performed tasks at both the first and fourth OTSS assessments are presented. Additionally, this table shows the proportion of facilities demonstrating improvement over these two assessment points. Just over half of laboratory facilities (21/38; 55.3%) demonstrated improvement over baseline OTSS assessments by correctly conducting an average of 4.8 additional tasks by the fourth OTSS assessment for observations of malaria blood slide and patient preparation. The largest gains for these observations were noted for patient/slide preparation (+ 1.4 tasks) and spreading thick films (+ 2.3 tasks). Similarly, an average of 5.4 additional, recommended tasks were correctly conducted by the fourth OTSS assessment among those laboratories (30/43; 69.8%) that improved performance during observations of malaria blood slide staining and reading. For these observations, laboratory technicians most improved performance for slide reading (+ 1.4 tasks) and reporting results (+ 1.3 tasks). Three quarters of assessed laboratories (31/41; 75.6%) that improved RDT use observation scores correctly completed an average of 6.0 additional RDT-specific tasks from the first to fourth OTSS assessments with largest gains in RDT preparation (+ 1.8 tasks) and patient preparation (+ 1.6 tasks) procedures. Among those clinics (36/58; 62.1%) demonstrating improvements in fever case management, assessed staff correctly performed an average of 3.3 additional tasks by the fourth OTSS assessment where health workers primarily improved upon providing patients with information regarding their diagnosis and treatment plan (+ 1.1 tasks).

**Table 1:** Mean number of tasks correctly performed during initial OTSS observations, assessment 1 vs. assessment 4

	Total tasks	Mean tasks correctly performed at assessment 1	Mean tasks correctly performed at assessment 4	Percent of facilities demonstrating improvement (%)
Preparation of blood films (n = 38 HFs)				
Patient/slide preparation	6	3.8	4.5	44.7
Specimen collection (finger prick)	5	4.1	4.0	23.7
Spreading thick films	5	3.1	4.0	42.1
Labelling	1	0.8	0.9	18.4
Disposal of infectious materials	2	1.9	2.0	10.5
Slide storage	1	0.6	0.9	34.2
Overall performance	20	14.3	16.3	55.3
Staining and reading blood films (n = 43 HFs)				
Preparation of Giemsa solution	3	2.3	2.7	30.2
Staining (Giemsa)	4	3.2	3.7	34.9
Slide drying	1	0.8	1.0	18.6
Slide examination	4	3.0	3.1	32.6
Slide reading	4	2.1	3.0	48.8
Result reporting	4	2.2	2.9	58.1
Result delivery	2	1.0	1.0	4.7
Overall performance	22	14.6	17.3	69.8
RDT procedures (n = 41 HFs)				
RDT preparation	6	4.1	5.2	51.2
Patient preparation	5	2.9	4.0	63.4
Blood collection and dispensing	5	4.1	4.4	41.5
RDT procedure and reading results	5	3.7	4.5	51.2
Recording results	2	1.6	1.8	31.7
Disposal of infectious material	2	1.8	2.0	17.1
Result delivery	2	0.9	1.0	9.8
Overall performance	27	19.1	22.9	75.6
Fever case management procedures (n = 58 HFs)				
Initial patient consultation (intake procedures)	4	3.2	3.3	31.0
Recording patient information and requesting tests	5	3.7	4.1	46.6
Interpretation of lab tests and prescribing medication	4	3.2	3.6	44.8
Provision of information to patient	4	2.8	3.2	44.8
Overall performance	17	13.0	14.2	62.1

*HF* health facility

Malaria diagnostic and clinical performance scores for RDT use, blood slide preparation, blood slide staining/reading, fever case management, and prescriber adherence to negative diagnostic test results were found to be small, but significantly improved from assessment 1 to assessment 4 via paired *t*-tests (Table 2). Gains in prescriber adherence performance were more apparent for health centres where scores increased by a mean of 11.9 percentage points (p = 0.04) than for hospitals which increased scores by a mean of 4.7 percentage points (p = 0.25) from OTSS assessment 1 to assessment 4. RDT use scores and blood slide preparation scores showed slight, but significant improvements from assessment 1 to assessment 2 with little to no overall change in scores from assessment 2 to assessment 3. Blood slide staining/reading scores showed no significant improvement from one assessment to the next; however, overall performance gains from assessment 1 to assessment 4 matched those for RDT use scores and blood slide preparation scores. Fever case management scores dropped 7 percentage points from the first to the second assessment, but increased thereafter by about 7 percentage points from the second to third and again from the third to the fourth assessments. Additionally, there was a significant reduction in the number of laboratories experiencing stock-outs of microscopy reagents/consumables and a significant increase in the number of laboratories instituting recommended laboratory practices such as the use of microscopy positive controls, conducting parasite counting, and converting from a semi-quantitative to a quantitative methodology (i.e. switching from the plus system of parasite quantification to parasites/µl) (Table 3). A non-significant reduction in RDT stock-outs and a non-significant increase in the number of facilities conducting slide rechecking as an IQA measure were also observed. Significant changes were also noted for the number of clinics properly maintaining both inpatient and outpatient registers. The internal process of re-checking malaria slides was observed in 38.8% (19/49) of hospital laboratories by the time of the fourth OTSS assessment up slightly from 33.3% (13/39) at baseline (p = 0.1356); no sustained increase or decrease in the percent of laboratories performing this IQA activity was noted for health centers. Among health centres, reported stock-outs of consumables that directly impeded the ability to perform malaria microscopy declined from 75.9% (22/29) at the first assessment to 24.1% (7/29) at the time of the fourth assessment (p < 0.0001). Hospitals experienced a similar trend in reported stock-outs declining from 51.9% (27/52) to 26.9% (14/52) from the first to fourth assessment (p = 0.0080).

**Table 2:** Performance trends in malaria diagnostic and clinical case management processes, assessment 1 vs. assessment 4

	OTSS assessment 1 vs. assessment 2			OTSS assessment 2 vs. assessment 3			OTSS assessment 3 vs. assessment 4			OTSS assessment 1 vs. assessment 4		
	HF No.	Ref (%)	Mean Diff	HF No.	Ref (%)	Mean Diff	HF No.	Ref (%)	Mean Diff	HF No.	Ref (%)	Mean diff
Malaria diagnostics												
RDT use	28	74.0	9.6**	36	80.8	0.5	52	80.5	7.2**	43	74.1	14.3***
Blood slide preparation	45	68.4	9.1**	44	76.4	0.3	51	76.0	4.0	51	65.2	14.7***
Blood slide staining/reading	50	69.0	0.7	46	69.0	2.3	50	74.1	4.3	51	66.3	14.0***
Clinical case management												
Fever case management	51	77.5	− 7.0**	50	69.6	*6.9	57	76.0	7.6**	57	76.2	7.3**
Prescriber adherence^a^	18	68.3	11.6	19	80.9	− 3.8	60	70.6	14.0**	61	77.2	7.2*

*HF* health facility, *No* number, *Ref* reference (beginning score for each *t* test scenario), *Mean Diff* mean differences from paired t-tests were calculated to characterize performance gains in mean scores from one assessment to the next and from OTSS assessment 1 to assessment 4

*** p < 0.001; ** p < 0.01; * p < 0.05

^a^Due to logistical issues during the 2nd OTSS assessment, prescriber adherence was minimally evaluated by clinical supervisors

**Table 3:** Change in selected health facility characteristics, assessment 1 vs. assessment 4

	HF no.	Assessment 1 HFs with factor		Assessment 4 HFs with factor		Exact p-value^a^
		n	%	n	%	
Laboratory characteristics/attributes						
Stock-outs of malaria microscopy consumables	81	49	60.5	21	25.9	*<* *0.001*
Stock-outs of RDTs	86	19	22.1	10	11.6	0.078
Uses microscopy positive controls	62	21	33.9	35	26.5	*0.003*
Conducts slide rechecking	60	17	28.3	23	38.3	0.308
Conducts species identification	61	12	19.7	13	21.3	1.000
Conducts parasite quantitation	62	37	59.7	50	80.6	*0.015*
Uses “plus system” for parasite quantitation	45	29	64.4	11	24.4	*<* *0.001*
Clinic characteristics/attributes						
Properly maintains outpatient registers	56	34	60.7	46	82.1	*0.012*
Properly maintains inpatient registers	46	32	69.7	42	91.3	*0.021*
Properly maintains clinical reports	41	35	85.4	40	97.6	0.125
Properly maintains drug stock cards	56	53	94.6	56	100.0	0.250

Statistically significant results are in italics

*HF* health facility, *No* number

^a^McNemar’s test of significance

### GLS linear regression

Tables 4, 5, 6 show the results of the bivariate analyses as well as the random effects GLS linear regression models fitted to predict determinants of fever case management performance (model 1), malaria blood slide staining/reading performance (model 2), and RDT use performance (model 3). The three dependent variables as well as observation scores included as covariates in the regression models are all expressed as percentages ranging from 0 to 100%. Number of OTSS visits is a categorical variable with 1, 2, 3, and 4 as the possible number of visits. Periodicity of OTSS visits is measured as completing all four assessments within 1 year vs. completing all four visits in a period of over 1 year. All other independent variables were coded as 1 or 0 depending on the presence or absence of equipment, stocks, and practices.

**Table 4:** GLS linear regression: clinician fever case management scores

	Unadjusted	Model 2 (adjusted)^a^
	Percentage point change (95% CI)	Percentage point change (95% CI)
Covariates		
Prescriber adherence to negative malaria tests (score)	*0.2* [*0.1*, *0.2*]***	*0.1* [*0.0*, *0.2*]**
Has ≥ 1 otoscope		
Yes	*5.4* [*1.3*, *9.6*]*	*4.4* [*0.0*, *8.7*]*
No	Reference	Reference
Has ≥ 1 ophthalmoscope		
Yes	*4.7* [*0.6*, *8.8*]*	
No	Reference	
Has ≥ 1 medical pen light		
Yes	*6.7* [*2.7*, *10.6*]**	4.0 [− 0.2, 8.2]
No	Reference	Reference
Has properly completed/maintained outpatient register		
Yes	2.1 [− 2.4, 6.6]	
No	Reference	
Has properly completed/maintained inpatient register		
Yes	*4.2* [− *0.7*, *9.0*]^†^	− 1.5 [− 6.6, 3.6]
No	Reference	Reference
Has properly completed/maintained clinical reports		
Yes	*8.4* [*2.2*, *14.5*]**	4.0 [− 2.6, 10.5]
No	Reference	Reference
Has properly completed/maintained drug stock cards		
Yes	*11.4* [*1.9*, *20.9*]*	1.8 [− 7.3, 10.8]
No	Reference	Reference
Has malaria case management and treatment guidelines		
Yes	*10.5* [*6.2*, *14.8*]***	*8.5* [*4.1*, *13.0*]***
No	Reference	Reference
Had an RDT stock-out of ≥ 7 days in the 3 months preceding OTSS visit		
Yes	*5.9* [− *0.8*, *12.6*]^†^	3.5 [− 3.4, 10.5]
No	Reference	Reference
Had a malaria microscopy consumables/reagents stock-out of ≥ 7 days in the 3 months preceding OTSS visit		
Yes	− *5.4* [− *9.5*, − *1.3*]*	− 1.0 [− 5.4, 3.3]
No	Reference	Reference
Periodicity of OTSS assessments		
All assessments occurred within a 1 year period	− 4.4 [− 10.7, 1.9]	
All assessments occurred in > 1 year period	Reference	
Number of OTSS assessments		
1	Reference	Reference
2	− *5.9* [*−* *10.8*, *−* *0.9*]*	− 2.4 [− 8.5, 3.7]
3	− 0.8 [− 5.8, 4.1]	0.0 [− 5.8, 5.8]
4	*6.0* [*1.1*, *11.0*]*	5.1 [− 0.9, 11.0]

Statistically significant results are in italics

*CI* confidence interval

Model 1: Wald X2 = 68.7 (p < 0.0001); 196 observations

*** p < 0.001; ** p < 0.01; * p < 0.05; ^†^ p < 0.1

^a^An ex-ante pairwise correlation test was run to detect collinearity; covariates indicating problematic correlation coefficients (≥ 0.55) were not included in the fully adjusted model

**Table 5:** GLS linear regression: malaria microscopy blood slide staining and reading observation scores

	Unadjusted	Model 2 (adjusted)^a^
	Percentage point change (95% CI)	Percentage point change (95% CI)
Covariates		
RDT observation score	*0.5* [*0.4*, *0.7*]***	
Malaria blood slide preparation observation score	*0.6* [*0.5*, *0.7*]***	*0.4* [*0.3*, *0.5*]***
All SOPs for malaria microscopy are present		
Yes	Reference	
No	− 5.5 [− 12.8, 1.8]	
All bench aids for malaria microscopy are present		
Yes	Reference	
No	− 0.6 [− 7.5, 6.3]	
Conducts malaria species identification		
Yes	*6.5* [*1.2*, *11.9*]*	3.7 [− 1.8, 9.2]
No	Reference	Reference
Uses parasites/µL for parasite quantitation		
Yes	*7.2* [*2.6*, *11.8*]**	− 0.4 [− 6.1, 5.3]
No	Reference	Reference
Uses “plus system” for parasite quantitation		
Yes	− *13.3* [− *17.3*, − *9.4*]***	− *7.2* [− *12.2*, − *2.1*]**
No	Reference	Reference
Uses microscopy positive controls		
Yes	*7.4* [*2.4*, *12.3*]**	3.7 [− 0.9, 8.3]
No	Reference	Reference
Stores stained microscopy slides for cross-checking		
Yes	*4.5* [*0.2*, *8.8*]*	2.0 [-2.7, 6.6]
No	Reference	Reference
Stock-outs of malaria microscopy consumables		
Yes	− 3.2 [− 7.3, 0.9]	
No	Reference	
Stock-outs of RDTs		
Yes	− 3.6 [− 9.5, 2.3]	
No	Reference	
Periodicity of OTSS assessments		
All assessments occurred within a 1 year period	*12.3* [*4.9*, *19.7*]**	*8.7* [*2.7*, *14.7*]**
All assessments occurred in > 1 year period	Reference	Reference
Number of OTSS visits		
1	Reference	Reference
2	1.2 [− 3.7, 6.1]	− 3.1 [− 9.4, 3.2]
3	*5.6* [*0.7*, *10.4*]*	− 2.7 [− 8.7, 3.4]
4	*11.0* [*6.1*, *15.8*]***	− 0.2 [− 6.5, 6.1]

Statistically significant results are in italics

*CI* confidence interval, *SOP* standard operating procedure

Model 2: Wald X^2^ = 96.8 (p < 0.0001); 183 observations

*** p < 0.001; ** p < 0.01; * p < 0.05

^a^An ex-ante pairwise correlation test was run to detect multicollinearity; covariates indicating problematic correlation (≥ 0.55) were not included in the fully adjusted model

**Table 6:** GLS linear regression: RDT use observation scores

	Unadjusted	Model 2 (adjusted)^a^
	Percentage point change (95% CI)	Percentage point change (95% CI)
Covariates		
Malaria blood slide preparation observation score	*0.5* [*0.4*, *0.6*]***	*0.5* [*0.4*, *0.6*]***
Malaria blood slide staining/reading score	*0.4* [*0.3*, *0.5*]***	
All SOPs for malaria microscopy are present		
Yes	Reference	
No	− 2.9 [− 8.9, 3.2]	
All bench aids for malaria microscopy are present		
Yes	Reference	
No	3.5 [− 2.1, 9.0]	
Conducts malaria species identification		
Yes	1.6 [− 3.2, 6.3]	
No	Reference	
Uses parasites/µL for parasite quantitation		
Yes	3.4 [− 0.7, 7.5]	
No	Reference	
Uses “plus system” for parasite quantitation		
Yes	− *6.7* [− *10.6*, − *2.8*]**	0.1 [− 4.1, 4.2]
No	Reference	Reference
Uses microscopy positive controls		
Yes	2.2 [− 1.7, 6.0]	
No	Reference	
Stores stained microscopy slides for cross-checking		
Yes	− 1.2 [− 5.0, 2.6]	
No	Reference	
Stock-outs of malaria microscopy consumables		
Yes	− *3.2* [− *6.7*, *0.2*]^†^	1.1 [− 2.8, 5.1]
No	Reference	Reference
Stock-outs of RDTs		
Yes	0.5 [− 4.9, 5.9]	
No	Reference	
Periodicity of OTSS assessments		
All assessments occurred within a 1 year period	*9.4* [*2.9*, *16.0*]**	4.9 [− 0.9, 10.7]
All assessments occurred in > 1 year period	Reference	Reference
Number of OTSS visits		
1	Reference	Reference
2	*4.8* [*0.4*, *9.1*]*	1.1 [− 4.7, 6.9]
3	*4.3* [*0.4*, *9.0*]*	1.3 [− 4.5, 7.1]
4	*9.5* [*5.1*, *13.7*]***	4.9 [− 1.1, 10.9]

Statistically significant results are in italics

*CI* confidence interval

Model 3: Wald X^2^ = 90.7 (p < 0.0001); 191 observations

*** p < 0.001; ** p < 0.01; * p < 0.05; ^†^ p < 0.1

^a^An ex-ante pairwise correlation test was run to detect multicollinearity; covariates indicating problematic correlation (≥ 0.55) were not included in the fully adjusted model

Model 1 was constructed to characterize possible determinants of case management performance during clinical consultations for patients with fever. Because independent emphasis was placed on prescriber adherence to negative test results outside of case management observations, prescriber adherence was included as a covariate in the overall model. Bivariate analysis showed that presence of certain medical equipment (otoscopes, ophthalmoscopes, and medical pen lights), properly maintained clinical documents (inpatient registers, clinical reports, and drug stock cards), presence of malaria case management and treatment guidelines, and prescriber adherence scores were all positively and significantly associated with fever case management performance score. Stock-outs of malaria microscopy consumables/reagents were negatively and significantly associated with this outcome. Having had two OTSS assessments was negatively associated with fever case management scores, but having had four OTSS assessments was positively associated with these scores. In the adjusted model, prescriber adherence scores, presence of at least one otoscope, and presence of malaria case management and treatment guidelines was found to be positively and significantly associated with clinician fever case management performance.

Models 2 and 3 explore determinants of malaria diagnostic performance related to blood slide staining/reading and RDT use, respectively. Bivariate analysis for microscopy blood slide staining/reading revealed two basic trends: improvements to other observed and scored malaria diagnostic techniques and the adoption/use of good laboratory practices, all of which were strongly promoted by the OTSS programme, had positive and significant associations with blood slide staining and reading. Additionally, facilities where all four OTSS assessments were conducted within a 1 year period and facilities with three and four OTSS visits were associated with significant gains in microscopy staining/reading performance scores. However, use of the “plus system” method of parasite quantitation—an imprecise method that entails using a code of between one and four plus signs to designate parasite density—showed a negative association under bivariate analysis reducing microscopy staining/reading performance scores by 13.3 (95% CI − 17.3 to − 9.4) percentage points. Under the adjusted model, blood slide preparation scores showed a positive and significant association with blood slide staining/reading scores. Additionally, laboratory facilities that used the “plus system” method of parasite quantitation as part of their microscopy procedures were found to have blood slide staining/reading observation scores that were 7.2 (95% CI − 12.2 to − 2.1) percentage points lower than those facilities not using this system. Facilities where all four OTSS assessments occurred within a 1 year period also had blood slide reading/staining scores that were 8.7 (95% CI 2.7–14.7) percentage points higher than facilities where the four assessments took over 1 year to complete. Bivariate analysis of RDT use observation scores showed that for every 1 percentage point improvement to malaria microscopy slide preparation and staining/reading scores, RDT use scores were increased by 0.5 (95% CI 0.4–0.6) and 0.4 (95% CI 0.3–0.5) percentage points, respectively. Similar to blood slide staining/reading scores, completing all four OTSS assessments within a 1 year period was positively associated with RDT use scores while technician use of the “plus system” was negatively associated with RDT use scores under bivariate analysis. Additionally, facilities with two, three, and four OTSS visits had slight, but positive and significant gains in RDT use scores compared to facilities with only one visit. Only blood slide staining/reading observation scores were significantly associated with RDT use observation scores under the adjusted model.

## Discussion

Similar to previous work [25–28], the results of this study show that supportive supervision may lead to improvements in malaria diagnostic performance. Item and bivariate analyses suggest that the OTSS programme was effective at improving microscopy and RDT diagnostic performance at the majority of facilities enrolled in the programme. While assessing different components of malaria case management, the results parallel those of Bello et al. [16] in that clinical staff targeted for supportive supervision showed small improvements to fever case management practices. Additionally, clinicians working within OTSS-enrolled health facilities demonstrated modest, but significant improvements with respect to their anti-malarial prescription practices following negative malaria test results. Further, regression analyses helped to contextualize observed gains exhibited among both clinicians and laboratory technicians by revealing specific determinants of overall performance. There were four broad categories of covariates included in the models: (1) covariates related to availability of specific resources or that might serve as proxy measures for overall availability of resources, (2) adoption of recommended practices, (3) scored laboratory performance in conducting malaria diagnostic tests, and (4) intensity and number of OTSS visits. An intended regression model that could not ultimately be completed due to small sample size was the effect of laboratory diagnostic performance on clinician fever case management performance improvements with respect to their anti-malarial prescription practices following negative malaria test results.

The presence of appropriate malaria case management and treatment guidelines was found to be a determinant of adherence to clinical algorithms for fever case management (regression model 1). These guidelines were available in the majority of clinics prior to the rollout of OTSS and part of the OTSS process was to ensure fever case management practices were aligned with the guidance prescribed within these documents. Through direct observation of febrile patient consultations, OTSS supervisors were able to address issues noted throughout the consultation process by making reference to available guidelines. Presumably, facilities without guidelines were at a disadvantage in that they were unable to make reference to proper case management and malaria treatment procedures in the wake of direct observation and subsequent corrective action. This finding could not be further contextualized; however, as Brugha et al. [23] have discussed, dissemination of clinical case management guidance is necessary, but not sufficient as a standalone intervention. Improvements to prescriber adherence scores, which increased by a mean of 14.0 percentage points from assessment 1 to assessment 4, were positively and significantly associated with small improvements to fever case management scores. Presence of otoscopes, medical pen lights, and properly maintained inpatient registers and clinical reports were all significant determinants of fever case management performance under bivariate analysis. It could be the case that behaviours compelling the acquisition of certain diagnostic tools or systematic (and correct) logging of patient information are simply characteristics of higher performing staff. Alternatively, the presence of diagnostic tools and properly maintained documents could be an indicator of external support and/or training or even serve as a proxy indicator for the overall availability of resources within the health facility itself. These are merely hypotheses as the study data set did not allow for person-level analysis, which may have elucidated additional determinants of clinician performance with respect to management of febrile patients.

Adoption of certain laboratory practices were important determinants of microscopy performance particularly for the bivariate analysis of model 2. Individuals working within facilities that conducted species identification as part of their malaria microscopy diagnostic procedures, used microscopy positive controls, and which stored stained microscopy slides for cross-checking demonstrated microscopy performance gains with respect to their slide staining/reading observation scores. Performance of these specific practices infer a willingness to institute or recommit to best practices as well as IQA measures within the laboratory. Implementation of laboratory IQA measures potentially constitute long-term solutions for sustained diagnostic performance. Species identification was not a commonly practiced component of malaria microscopy likely due to the fact that most malaria infections in sub-Saharan Africa are cause by *Plasmodium falciparum* [32]. However, those facilities conducting species identification, a relatively difficult skill to acquire, demonstrated higher scores for malaria microscopy staining/reading. Additionally, as many laboratory technicians performed both RDTs and microscopy and received focused on-the-job training for both test types, performance gains in one were expected to coincide with gains in the other. Indeed, for every point increase in RDT use performance about a half point increase in malaria slide staining/reading performance was observed and vice versa. The periodicity of OTSS visits was a covariate particularly indicative of improved performance for malaria blood slide staining/reading. Under the adjusted model, laboratories with approximately quarterly visits demonstrated blood slide staining/reading scores 8.7 percentage points higher than those laboratories where OTSS visits were completed in greater than a 1 year period.

Clinician ACT prescription practices with respect to adherence to malaria parasitological test results have a clear impact on patient health outcomes. Even slight performance gains in prescriber adherence to negative malaria test results are of great importance because the health and well-being of effected patients are directly impacted. Patients diagnosed with malaria based on clinical findings alone may be prescribed an ACT when in fact the underlying cause of their symptoms is unrelated to malaria. Improving the capacity of clinicians to conduct patient consultations in line with fever case management protocols and associated treatment algorithms via supportive supervision has, at least in one study, increased reliability on malaria parasitological results for final patient diagnosis [16]. Similarly, providing regular malaria RDT and microscopy on-the-job training opportunities to health facility diagnosticians may serve to increase clinician confidence in the test results they produce or receive. Establishing IQA and good laboratory practices to ensure reproducibility and sensitivity of diagnoses in addition to a system of routine malaria slide cross-checking may improve clinician confidence in overall laboratory practices and thus specific test results. To this end, the OTSS programme paired clinical and laboratory supervisors who conducted their supervisory visits in tandem with an expressed intent of fostering communication across departments. However, the OTSS programme did not explicitly monitor or evaluate connections between clinical and laboratory departments of participating health facilities. Future work focusing on improvements to diagnostic accuracy and clinical case management of malaria should strive to strengthen or foster lines of communication between laboratory and clinical staff.

There were a number of limitations to this work. All of the results presented in this paper need to be considered in the absence of a control group. Additionally, the OTSS programme was observational in nature making it difficult to isolate the effects of supportive supervision. Moreover, it may not be entirely possible within an EQA framework that relies on direct observation of facility staff by senior level biological scientists and clinicians to conduct these observations without some degree of observer bias. However, this phenomenon was minimized in certain instances as OTSS teams partially relied on record reviews to confirm specific practices (e.g. prescriber adherence). Additionally, health facility institutionalization of good laboratory practices and microscopy-specific IQA processes could be a consequence of the OTSS programme as supervisors promoted these measures at each assessment primarily to bring facilities in line with previously established guidance.

The OTSS programme relied on direct observations of laboratory staff in order to assess performance and document changes in RDT and malaria microscopy practices over time. Individual procedures against which laboratory technicians were evaluated were comprised of multiple tasks. OTSS teams were only required to record the total number of tasks correctly completed for each procedure thereby limiting the ability to comprehensively analyse areas of strength/weakness. However, the OTSS programme documented improvement to critical categories of malaria diagnostic procedures including spreading and reading thick films—two areas essential for correct interpretation of slide results. One possible alternative to the approach the OTSS programme used to evaluate performance would be to streamline the task lists to only include those elements considered essential to obtaining highest quality outcomes with respect to correct interpretation of test results. This would ensure that supervisors could effectively balance the need to critically observe staff for corrective action and make record of their performance at the same time. The EQA teams initially advocated for streamlining the observation process, but met resistance from senior OTSS supervisors who made the argument that reducing or minimizing versions of internationally recognized standard operating procedures was not in the best interest of their country’s malaria programme.

It is possible that gains in clinical case management and diagnostic performance might be attributable to some mechanism external to the OTSS programme. Indeed, secular changes in prescriber adherence practices as a result of increasing familiarity with and acceptance of RDT results could have contributed to observed trends. The relatively limited timeframe in which the OTSS assessments occurred, however, may have constrained the overall impact of longer-term secular effects. Additionally, the OTSS programme was the only one of its kind and intensity being administered to the enrolled health facilities throughout the duration of the IMaD project. The extrapolation of results to other facilities in general is cautioned as the sample was not selected randomly from a pool of eligible facilities (i.e. facilities with functional laboratories and higher patient throughput).

There are other important qualitative and quantitative measures that could be taken into account to more fully realize the impact of supportive supervision programmes. Key informant interviews and focus group discussions with NMCP representatives, OTSS supervisors, health facility administrators, and members of the health workforce could be conducted to better understand the mechanistic properties of OTSS that cannot be captured through supervision checklists alone. Such qualitative work could reveal a set of best practices and effective approaches for establishing working mentor–mentee relationships, methods for the resolution of identified performance issues, how best to optimize the allocation of resources as well as procurement/forecasting strategies, and instituting processes for internal quality improvement of health facility processes. Qualitative research could also help to describe changes to clinician case management behaviours with respect to performance gains in laboratory diagnostics for malaria. Another component that future implementers may consider exploring is the concept of diminishing returns regarding laboratory and clinical staff performance gains following multiple rounds of supervisory assessments. Additionally, an economic evaluation is needed to determine if a programme such as OTSS is worth continuing compared to alternative programmes that could be implemented with the same resources.

## Conclusion

The OTSS programme served as an effective performance appraisal system embedded within the health system and helped to ensure that staff had the right competencies to successfully complete their work. This programme suggests that supportive supervision with focused capacity building may lead to modest improvements to malaria diagnostic skills, adoption of laboratory best practices including components of IQA, strengthened fever case management practices, and improved prescriber adherence to negative malaria test results. Further investigation is needed to elucidate the relationship between laboratory diagnostic performance and its effects on clinician prescribing practices. Future work may focus on strengthening the clinic-laboratory interface within health facility departments to ensure gains made in laboratory performance are well understood and taken into consideration by clinical staff.

## Additional file

**Additional file 1.** Laboratory and clinical checklists for observations.

## Abbreviations

ACT: artemisinin-based combination therapy
GLS: generalized least squares
IMaD: Improving Malaria Diagnostics project
IQA: internal quality assurance
NMCC: National Malaria Control Centre
OTSS: outreach training and supportive supervision
RDT: rapid diagnostic test
WHO: World Health Organization
