# Supplementary material for: Outreach training and supportive supervision for malaria case management in Zambia: the effects of focused capacity building on indicators of diagnostic and clinical performance
Source: Malar J. 2018 Nov 28;17:438. doi: 10.1186/s12936-018-2589-6 (PMC6260723; doi:10.1186/s12936-018-2589-6)
Supplement: Supplementary file 1 — Additional file 1. Laboratory and clinical checklists for observations. [file 12936_2018_2589_MOESM1_ESM.docx]

Additional file 1

**Preparation of Thick Blood Films**

| Patient/slide preparation (6 tasks) |
| --- |
| 1. Technician washed his/her hands 2. Technician verified the request form for the test (has consulted with health worker, if required), date and time the request was received was recorded 3. Patient identified; patient’s details and laboratory number recorded 4. Test procedure explained to patient; reassurance provided as needed 5. Technician wore gloves 6. The microscopy slide was cleaned/wiped before use; slide was handled only by its edges |
| Specimen collection – finger prick (5 tasks) |
| 1. Puncture site was selected, cleaned with alcohol swab, and allowed to dry 2. Puncture site was firmly pricked with sterile lancet 3. Technician did not excessively squeeze finger 4. First drop of blood wiped off, next drop(s) of blood deposited onto slide without the finger touching the slide 5. Pressure applied to puncture site with cotton wool |
| Spreading thick film (5 tasks) |
| 1. Spreader was clean and had smooth surface/edge 2. Only one patient sample per slide 3. Thick film measured about 1cm in diameter; print/text can be read through the blood smear 4. Slide placed horizontally to air dry before staining; no heating to dry film 5. Slide was protected against dust, flies, etc. as it dried |
| Labeling (1 task) |
| 1. Slide/film was labeled with patient’s laboratory number and date |
| Disposal of infectious materials (2 tasks) |
| 1. Technician disposed of lancet, needle/syringe, or vacutainer tube into sharps/biohazard container 2. Technician discarded other contaminated materials into lined container |
| Slide storage (1 task) |
| 1. Slide(s) cleaned of oil/grease and properly stored as required for reuse or quality assurance |

**Staining and Reading Thick Blood Films**

| Preparation of stain (3 tasks) |
| --- |
| 1. Giemsa stain stock solution was labeled with time and date, kept well-stoppered, and filtered regularly to remove precipitate 2. Giemsa stock diluted to 10% with a buffer of pH between 7.2 - 7.4, discarded after 6-8 hours 3. If Giemsa solution is scarce and re-used, the solution was filtered |
| Staining – Giemsa stain (4 tasks) |
| 1. If the slide has a thin film, only the thin film is fixed with absolute methanol (2-3 seconds) and dried before staining with Giemsa 2. Slide was flooded with Giemsa for 10-15 minutes 3. Slide was removed from the stain and rinsed with clean water 4. Slide was drained and dried on a rack, no heating to dry slide |
| Slide drying (1 task) |
| 1. Slides were drained and dried on an incline on a drying rack; no heating to dry slides |
| Slide examination (4 tasks) |
| 1. Technician placed immersion oil on slide 2. Rack stage of microscope was lowered before slide positioned on stage 3. Technician focused with 100x oil immersion objective 4. Technician examined ≥ 100 fields before declaring the slide negative (about 10 minutes) |
| Slide reading (4 tasks) |
| 1. Technician differentiated negative from positive result 2. Technician attempted species and stage identification 3. Technician attempted parasite quantification by counting 200 – 500 WBC 4. If parasite density is high, thin film used for counting |
| Result reporting (4 tasks) |
| 1. Technician reported negative or positive for parasites 2. Technician reported species and stage(s) of parasite(s) 3. Technician reported parasite quantification per 200 – 00 WBC or conversion to parasites/µL (as required) 4. Technician recorded date and time of reporting result |
| Result delivery (2 tasks) |
| 1. Technician delivered results to patient and/or clinician 2. Time taken from receiving test request to results delivery is noted |

**Rapid Diagnostic Test (RDT)**

| RDT preparation (6 tasks) |
| --- |
| 1. RDTs stored at < 30°C 2. RDTs stored away from direct sunlight 3. RDTs stored away from windows 4. Tester verified expiration date 5. Tester used test kit with earliest expiration date 6. RDT cassette was at room temperature before tester began procedure |
| Patient preparation (5 tasks) |
| 1. Tester washed his/her hands 2. Tester verified the request form for the test (consulted with health worker, if required), date and time the request was received was recorded 3. Patient identified; patient’s details and laboratory number recorded 4. Test procedure explained to patient; reassurance provided as needed 5. Tester wore gloves |
| Blood collection and dispensing (5 tasks) |
| 1. Puncture site was selected, cleaned with alcohol swab, and allowed to dry 2. Puncture site was firmly pricked with sterile lancet 3. Tester did not excessively squeeze finger 4. Tester collected an adequate volume of blood 5. Tester dispensed blood sample in correct well |
| RDT procedure and reading results (5 tasks) |
| 1. Tester dispensed diluent in correct well 2. Tester dispensed the correct volume of diluent 3. Tester waited the correct amount of time for the test to run (according to manufacturer’s instructions 4. Tester verified internal test control 5. Tester read results correctly |
| Recording results (2 tasks) |
| 1. Results were correctly recorded (including mixed infection if a combo test was used) 2. Date and time results were reported is recorded |
| Disposal of infectious material (2 tasks) |
| 1. Used tests, fluid transfer devices, and other contaminated materials disposed of into lined container 2. Used lancets disposed of into a sharps/biohazard container |
| Result delivery (2 tasks) |
| 1. Results delivered back to patient and/or clinician 2. Time taken from receiving test request to results delivery was noted |

**Fever case management**

| Initial patient consultation (intake procedures) |
| --- |
| 1. Health worker makes the patient as comfortable as possible 2. Health worker asks relevant questions 3. Health worker carries out physical examination 4. Health worker uses appropriate diagnostic equipment correctly |
| Recording patient information and requesting tests |
| 1. Health worker records relevant information 2. Health worker records date and time of filling in diagnostic test request form 3. Health worker makes a diagnostic impression 4. Health worker requests relevant laboratory tests 5. Health worker receives results within reasonable time frame |
| Interpretation of lab tests and prescribing medication |
| 1. Health worker interprets laboratory investigations correctly 2. Health worker makes a correct diagnosis 3. Health worker prescribes appropriate treatment 4. Health worker prescribes drugs in appropriate doses and duration (according to national standard treatment guidelines) |
| Provision of information to patient |
| 1. Health worker explains findings and treatment plan to patient 2. Health worker gives patient clear instructions for convalescence 3. Health worker gives appropriate health education 4. Health worker arranges for follow-up, if necessary |
